# Supplementary material for: Pathways and processes to adopting or switching to a single-dose HPV vaccination schedule in low- and middle-income countries: a qualitative study
Source: PLOS Glob Public Health. 2025 Feb 5;5(2):e0004245. doi: 10.1371/journal.pgph.0004245 (PMC11798430; doi:10.1371/journal.pgph.0004245)
Supplement: Attachment — Submitted filename: ResponseMemo.docx [file pgph.0004245.s003.docx]

**Reviewer #1:**

The manuscript reports the findings of a study that aims to characterize the sequence of events, as well as actors/institutions involved, that facilitate or impede country-level decision-making related to the HPV vaccine schedule. With the new recommendations for countries to consider using single dose HPV vaccine by WHO and its SAGE and the high burden of cervical cancer in low and middle income countries, this is an important and valuable study to understand the influencing factors in countries being able to consider and introduce the new schedule.

The authors have systematically described the methods and process of analysis in good depth and clarity. Clarification on the ethical considerations are also made clear. Data is not made available but the authors have responded to the PLOS question on data availability regarding it being sensitive information and that they would be shared upon request in a de-identifying manner.

We thank the reviewer for their critical, positive appraisal of our manuscript.

Two things that the authors should consider and further clarify in their manuscript -

1. Although through interviews the study has been able to demonstrate the processes, key actors and agencies, and factors influencing the decision making, the study has limitations in not having explored the underlying political forces in operation which may remain unsaid in the interviews. The influence of lobby groups, pharmaceutical industry and other powerful agencies have over even the so-called “scientific” body or its recommendations. The idea that these processes are not merely scientific but also deeply political. Perhaps the authors can clarify this as one of their study limitations. The role of peripheral bodies are explored to a certain extent and further discussed in the discussion section but by design, the study hasn’t explored the political root causes/processes.

We thank the reviewer for this thoughtful point about the political determinants of these decision-making pathways/processes. We whole-heartedly agree that these processes are not only scientific but also inherently political and are aligned with the reviewer’s argument that our study design and sampling strategy may not have accommodated a thorough, robust investigation of these political determinants. At the reviewer’s suggestion, we have integrated discussion of these points into our Limitations paragraph, as follows:

*“Third, in-depth interviews with immunization stakeholders emphasized country-led pathways/processes for determining vaccination schedules, likely overlooking political determinants of these processes (e.g., influence of pharmaceutical companies and lobbying groups). Given that immunization schedule decision-making is both a scientific and political process, future studies should explicitly characterize the influence of political actors on pathways/processes to HPVV schedule-related decision-making in LMICs.”* (Pg. 18-19, Lines 361-367)

2. The current study team’s organization and study’s funding organization are key players in the networks through which the participants were recruited. This would have additional risk of participants withholding sensitive information and is a limitation that can be mentioned.

We thank the reviewer for also calling our attention to this limitation of our study and agree that the sampling strategy may have reinforced some of the sensitivities around this topic, especially considering the nature of the investigative team’s rapport with immunization stakeholders. Accordingly, we have added the following language to our Limitations:

*“Second, the sensitivity of HPVV decision-making, especially in countries that had yet rendered a decision on the immunization schedule, might have demotivated stakeholders from sharing or divulging specific details or information during the interviews;* ***the professional networks from which stakeholders were sampled may have further amplified sensitivities surrounding these interviews and prompted non-disclosure of specific HPVV pathways/processes by participants.****”* (Pg. 18, Lines 356-361)

**Reviewer #2:**

This manuscript examines immunization stakeholders’ roles in low- and middle-income countries (LMICs) to better understand how crucial the stakeholder groups’ HPV vaccine (HPVV) recommendations are after the WHO revised the HPVV schedule to support single dose vaccinations. The authors effectively outlined the need for this type of qualitative research by providing the reader with a clear understanding of why HPVVs are important, the benefit of single dose schedules, and how stakeholder groups (i. e. NITAGs) influence formal vaccine schedules.

We thank the reviewer for their thoughtful, positive appraisal of our work and critical review.

Having 66 participants interviewed allows for diverse representation. However, the authors should include the specific professional backgrounds/positions of the participants and the number of participants from each country, so the reader can note which countries had more interview participants. The methods section refers to the interview guide that included the participants’ professional roles, therefore these data should be provided in the manuscript to display stakeholder/participant qualifications (doctors, epidemiologists, etc.). Providing tables with where each participant was from and their positions would allow the reader to determine the diversity (or lack of) of the participant population. Providing these data should demonstrate reliability.

We thank the reviewer for these suggestions. While we agree that disseminating this information to readers would be important to further enhance the credibility of findings, we intentionally decided not to disclose this information, especially at country level, to protect the privacy and confidentiality of our participants, who in some instances may have been speaking about deeply sensitive and politically polarizing topics in their contexts. As such, we have continued to mask the identities of participants and only broadly present their institutional/organizational affiliations in the aggregate:

*“We conducted interviews with 66 national immunization stakeholders (MoH/EPI or NITAG/TWG members [n = 26], technical assistance partners or civil society organizations [n = 22], multilateral organizations [n = 18]) from 19 LMICs...”* (Pg. 10, Lines 159-161).

Nevertheless, we agree with the reviewer’s assessment that presenting the number of interviewers conducted per country would be helpful, and our revised manuscript now presents this information in text in the opening paragraph of the Results section:

*“We conducted interviews with 66 national immunization stakeholders (MoH/EPI or NITAG/TWG members [n = 26], technical assistance partners or civil society organizations [n = 22], multilateral organizations [n = 18]) from 19 LMICs,* ***11 of which had introduced HPVV before the publication of the WHO position paper on single-dose HPVV (Burkina Faso [n = 5], Cambodia [n = 3], Cote d’Ivoire [n = 4], Eswatini [n = 3], Ethiopia [n = 4], Indonesia [n = 3], Kenya [n = 5], Laos [n = 3], Philippines [n = 4], Tanzania [n = 5], Uganda [n = 2]), and 8 countries were classified as new HPVV introductions (Bangladesh [n = 3], Cameroon [n = 5], Ghana [n = 4], India [n = 2], Madagascar [n = 1], Nepal [n = 3], Nigeria [n = 3], Pakistan [n = 4])****.”* (Pg. 10, Lines 159-166)

Lastly, table 1 should be reviewed for consistent language in the “income status” column.

We thank the reviewer for calling our attention to some of these discursive inconsistencies in Table 1. These have now been rectified in our revised manuscript.

Overall, this manuscript will be a valuable contribution to existing STI prevention and public health/policy literature by outlining how NITAGs’ recommendations determine LMICs’ HPVV schedules and what resources (i.e. funding) impact those recommendations. This paper provides a sound analysis of NITAGs in LMICs and how important their role in global health is.

We thank the reviewer for their positive assessment of our manuscript and for articulating the promise of its prospective contributions to the field.
